# NEIL1 and NEIL2 Are Recruited as Potential Backup for OGG1 upon OGG1 Depletion or Inhibition by TH5487

**DOI:** 10.3390/ijms22094542

**Published:** 2021-04-27

**Authors:** Bishoy M. F. Hanna, Maurice Michel, Thomas Helleday, Oliver Mortusewicz

**Affiliations:** 1 Science for Life Laboratory, Department of Oncology-Pathology, Karolinska Institutet, 171 65 Stockholm, Sweden; bishoy.hanna@ki.se (B.M.F.H.); maurice.michel@ki.se (M.M.); thomas.helleday@ki.se (T.H.); 2 Weston Park Cancer Centre, Department of Oncology and Metabolism, University of Sheffield, Sheffield S10 2RX, UK

**Keywords:** NEIL1 glycosylase, NEIL2, OGG1 inhibitor, TH5487, DNA oxidative damage, 8-oxoguanine, base excision repair, backup pathway, recruitment kinetics, chromatin binding dynamics

## Abstract

DNA damage caused by reactive oxygen species may result in genetic mutations or cell death. Base excision repair (BER) is the major pathway that repairs DNA oxidative damage in order to maintain genomic integrity. In mammals, eleven DNA glycosylases have been reported to initiate BER, where each recognizes a few related DNA substrate lesions with some degree of overlapping specificity. 7,8-dihydro-8-oxoguanine (8-oxoG), one of the most abundant DNA oxidative lesions, is recognized and excised mainly by 8-oxoguanine DNA glycosylase 1 (OGG1). Further oxidation of 8-oxoG generates hydantoin lesions, which are recognized by NEIL glycosylases. Here, we demonstrate that NEIL1, and to a lesser extent NEIL2, can potentially function as backup BER enzymes for OGG1 upon pharmacological inhibition or depletion of OGG1. NEIL1 recruitment kinetics and chromatin binding after DNA damage induction increase in cells treated with OGG1 inhibitor TH5487 in a dose-dependent manner, whereas NEIL2 accumulation at DNA damage sites is prolonged following OGG1 inhibition. Furthermore, depletion of OGG1 results in increased retention of NEIL1 and NEIL2 at damaged chromatin. Importantly, oxidatively stressed NEIL1- or NEIL2-depleted cells show excessive genomic 8-oxoG lesions accumulation upon OGG1 inhibition, suggesting a prospective compensatory role for NEIL1 and NEIL2. Our study thus exemplifies possible backup mechanisms within the base excision repair pathway.

## 1. Introduction

It is estimated that tens of thousands of DNA bases are chemically modified in each cell per day [1]. The highly conserved base excision repair (BER) pathway is the major pathway for repairing DNA damage caused by oxidation, alkylation and deamination. Such aberrant DNA lesions cause no significant distortion to the DNA helical structure, yet they have to be efficiently repaired in order to maintain genomic integrity. BER is a multistep DNA repair process initiated by a DNA glycosylase that recognizes its DNA substrate lesion and cleaves the N-glycosidic bond between the base and the deoxyribose. Following base excision, other downstream enzymes are recruited to process the resulting abasic site, install an undamaged nucleotide and seal the nick in the DNA backbone [2].

When it comes to oxidative DNA damage, guanine is particularly prone to oxidation because of its low redox potential [3]. Guanine oxidation gives rise to a plethora of oxidation products, of which 7,8-dihydro-8-oxoguanine (8-oxoG) has been the most extensively studied lesion [4,5]. 8-oxoguanine DNA glycosylase 1 (OGG1) is the main glycosylase recognizing and excising 8-oxoG opposite to cytosine in duplex DNA [6,7]. Being a bifunctional enzyme, OGG1 possesses a DNA glycosylase activity in addition to a weak AP lyase activity [8,9]. Beyond its well-established role in BER, multiple lines of evidence suggest that OGG1 plays a role in modulating gene expression upon binding to 8-oxoG located in gene regulatory regions [10,11]. For instance, OGG1 interaction with 8-oxoG in guanine-rich promoters of proinflammatory genes facilitates NF-κB binding and subsequent proinflammatory gene expression [12,13].

The redox potential of 8-oxoG itself is approximately 600 mV below that of guanine. Consequently, 8-oxoG is extremely sensitive to further oxidation, giving rise to hydantoin lesions [14,15,16]. These DNA lesions are recognized by the Nei endonuclease VIII–like family of DNA glycosylases (NEIL). Three human NEIL enzymes have been identified, NEIL1, NEIL2 and NEIL3, and have been shown to efficiently repair spiroiminodihydantoin (Sp) and guanidinohydantoin (Gh) in addition to other lesions including thymine glycol, 2,6-diamino-4-hydroxy-5-formamidopyrimidine (FapyG) and 4,6-diamino-5-formamidopyrimidine (FapyA) [17,18,19].

In vitro assays have indicated that NEIL1 possesses a weak incision activity against 8-oxoG [20,21,22]. Using an in vitro model for clustered lesions, Parsons et al. reported that NEIL1 catalyzes the excision of 8-oxoG when located as a third or fourth nucleotide 5′-upstream to a single-strand break [23]. Furthermore, NEIL1 excises hydantoin lesions present in bubble, bulge and single-stranded DNA in in vitro assays [24]. Cellular studies have demonstrated that NEIL1 is involved in pre-replicative repair of oxidative lesions in single-stranded replicating DNA templates. Interestingly, NEIL1 was found to bind to, but not excise, the base lesion preventing the generation of lethal double-strand breaks. In doing so, NEIL1 blocks the progression of the replication fork, which then regresses to enable lesion repair [25,26]. Furthermore, excision of 5-hydroxyuracil and thymine glycol by NEIL1 increases within double-stranded DNA close to the replication fork junction, which can mimic a structure representing a regressed DNA replication fork [27]. Besides, NEIL1 is implicated in the excision of inter-strand cross links [28,29,30] as well as excision of hydantoin lesions from telomeric and promoter quadruplexes, thus contributing to telomere maintenance and gene regulation [31]. In addition, highly transcribed genomic regions are enriched in acetylated NEIL1. This preferential binding of acetylated NEIL1 correlates with low mutation frequencies, implying that NEIL1 protects transcription start sites from oxidative DNA damage [32]. Importantly, NEIL1 and NEIL2 share common substrates including FapyG, and FapyA, thymine glycol and 5-hydroxyuracil present in single-stranded, duplex and bubble-structure oligonucleotides [33]. Both NEIL1 and NEIL2 can recognize and excise Sp and Gh, the oxidation products of 8-oxoG [17]. While, NEIL1 has been reported to be involved in repairing oxidized lesions in replicating DNA [25], NEIL2 is thought to be preferentially involved in BER of oxidized lesions during transcription [34].

The overlapping roles of BER proteins became evident from knockout mice studies. In general, DNA glycosylase-deficient mice have been found to be notably resilient to the loss of glycosylase activities [2,35,36]. Except for thymine DNA glycosylase, knockout mice of a single DNA glycosylase are viable, showing only a moderate increase in mutation frequency but no overt disease phenotype [37,38]. However, double or triple knockouts targeting backup functions can display a strong phenotype. For instance, double knockout mice lacking both OGG1 and MUTYH (MutY homolog) are highly susceptible to cancer and have shortened life spans [39]. Of note, OGG1-deficient mice are viable and fertile. Despite the absence of a pathological phenotype, a 1.7–7-fold increase in the steady-state level of 8-oxoG was detected in nuclear DNA isolated from the liver of 13–15-week-old OGG1 null mice [40,41]. Intriguingly, no elevated spontaneous tumor incidence was detected in these mice although they showed a moderate increase in spontaneous mutation rate suggesting the presence of a backup repair pathway [40].

Whether backup mechanisms are activated once a specific glycosylase is inhibited has remained elusive due to the absence of specific small molecule glycosylase inhibitors. We previously reported the development of TH5487, an active site OGG1 inhibitor [13,42,43]. Here, we sought to analyze if NEIL1 or NEIL2 can function as backup BER enzymes for OGG1. We employed TH5487 as a tool compound to pharmacologically inhibit OGG1 and studied NEIL1 and NEIL2 recruitment kinetics as well as chromatin binding following OGG1 inhibition. We observe an increased recruitment of NEIL1 to DNA damage sites in addition to amplified NEIL1-chromatin binding upon DNA damage induction in TH5487-treated cells. Furthermore, NEIL2 accumulation at DNA damage sites is prolonged after OGG1 inhibition. Knockdown of OGG1 in oxidatively stressed cells results in increased 8-oxoG levels and increased retention of NEIL1 and NEIL2 at damaged chromatin, similar to what is observed upon treatment with TH5487. Importantly, more genomic 8-oxoG lesions accumulate upon induction of oxidative stress and concomitant OGG1 inhibition in NEIL1- or NEIL2-depleted cells. Our findings suggest a potential compensatory role for NEIL1, and to a lesser extent NEIL2, when OGG1’s function is compromised.

## 2. Results

### 2.1. OGG1 Inhibitor TH5487 Does Not Bind to NEIL1

**Figure 1:**
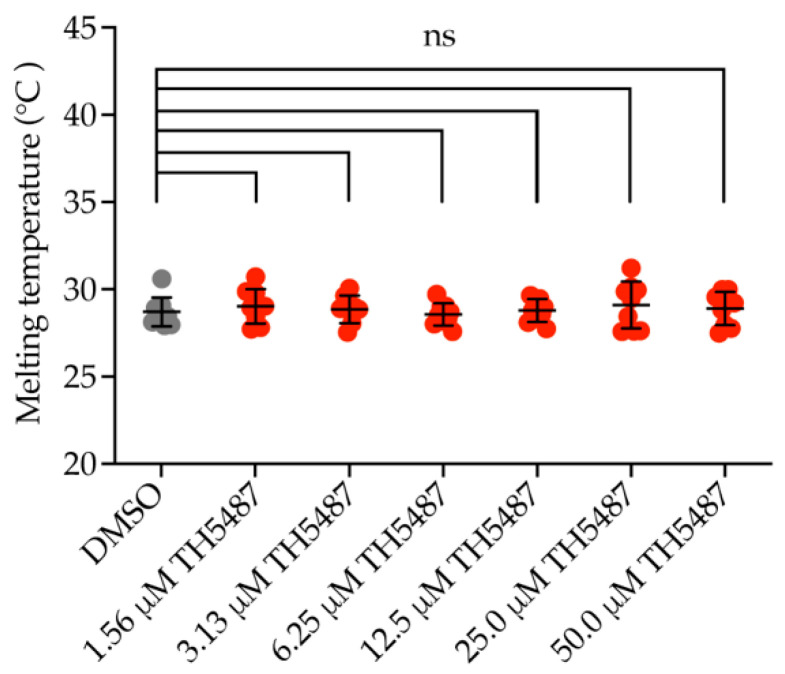
TH5487 does not interact with NEIL1. Differential scanning fluorimetry was useed to determine binding of NEIL1 to TH5487. NEIL1 was incubated with SYPRO Orange and a dilution series of OGG1 inhibitor, TH5487. Data are presented as mean ± SD of three technical replicates from three independent experiments. Statistical significance was determined using unpaired, two-sided *t*-test (ns, non-significant).

Previously, we reported the development of TH5487, a potent selective OGG1 inhibitor that binds OGG1’s active site, hampering its binding to DNA and causing accumulation of genomic 8-oxoG lesions in cells [13,42,43]. Since 8-oxoG is prone to further oxidation into spiroiminodihydantoin (Sp) and guanidinohydantoin (Gh) [14,15,16], we aimed to study if NEIL1 can function as a backup glycosylase after enzymatic inhibition of OGG1. Differential scanning fluorimetry (DSF) showed that TH5487 does not bind to NEIL1 (Figure 1), excluding off-target interaction of TH5487 with NEIL1. This is in agreement with previously reported in vitro biochemical assay results showing that TH5487 inhibits OGG1 but not NEIL1 [13].

### 2.2. TH5487 Treatment Results in Increased Recruitment of NEIL1-GFP to DNA Damage Sites

**Figure 2:**
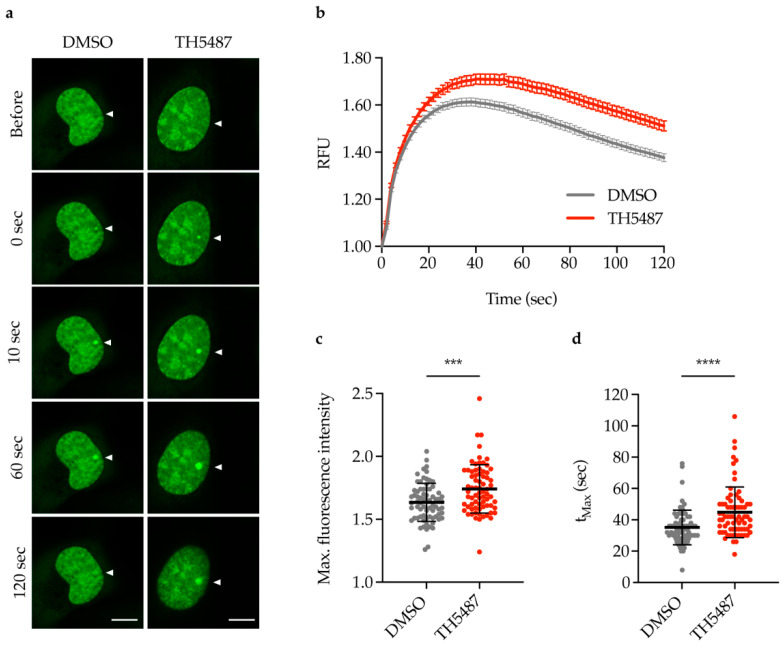
TH5487 treatment results in increased recruitment of NEIL1-GFP to DNA damage sites. (**a**) NEIL1 recruitment kinetics. U2OS cells expressing NEIL1-GFP were treated with either 0.1% DMSO or 10 μM TH5487 for 1 h, pre-sensitized with Hoechst, and irradiated with a 405 nm laser to induce DNA damage. Representative images before and after laser microirradiation at the indicated time points are shown. Arrows indicate the spots of laser irradiation. Scale bar, 10 μm. (**b**–**d**) Quantification of NEIL1-GFP recruitment kinetics (**b**), maximum fluorescence intensity (**c**) and time needed to reach maximum fluorescence intensity (**d**) in U2OS cells treated as described in (**a**). RFU, relative fluorescence units. Data are mean ± SEM (**b**) or mean ± SD (**c**,**d**) of six independent experiments. Statistical significance was determined using unpaired, two-sided *t*-test (*** *p* < 0.001, **** *p* < 0.0001).

Having excluded off-target interaction between TH5487 and NEIL1, we studied NEIL1 recruitment to DNA damage sites in TH5487-treated cells. Laser microirradiation experiments were performed in U2OS (osteosarcoma) cells stably expressing NEIL1-GFP following exposure to TH5487. TH5487 treatment resulted in increased and prolonged NEIL1 recruitment to laser-induced DNA damage sites (Figure 2a–d). These results indicate that NEIL1 could play a compensatory role in DNA repair when the enzymatic function of OGG1 is inhibited.

### 2.3. NEIL1-Chromatin Binding Increases upon DNA Damage Induction and TH5487 Treatment

**Figure 3:**
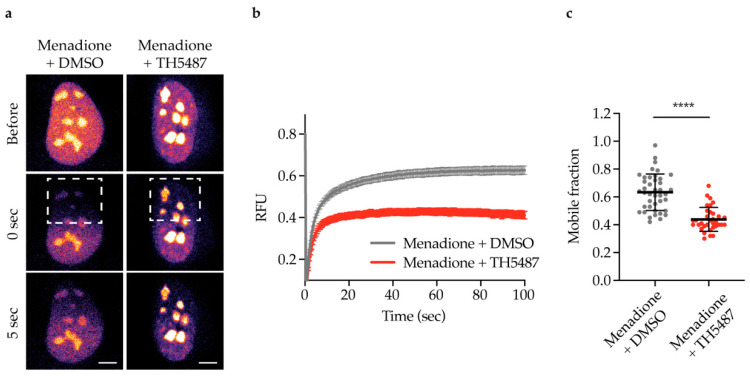
NEIL1-chromatin binding increases upon TH5487 treatment in oxidatively stressed cells. (**a**) Fluorescence recovery after photobleaching (FRAP) of U2OS cells expressing NEIL1-GFP co-treated with 50 μM menadione and either 0.1% DMSO or 10 μM TH5487 for 1 h. A nuclear region was bleached, and recovery of fluorescence after photobleaching was recorded. Dashed outlines indicate bleached areas. Representative false-color images of treated cells at the indicated time points are shown. Scale bar, 5 μm. (**b**,**c**) Quantification of NEIL1-GFP fluorescence recovery after photobleaching (**b**) and mobile fraction (**c**) in U2OS cells treated as described in (**a**). RFU, relative fluorescence units. Data are mean ± SEM (**b**) or mean ± SD (**c**) of three independent experiments. Statistical significance was determined using unpaired, two-sided *t*-test (**** *p* < 0.0001).

If NEIL1 functions as a backup glycosylase for OGG1, NEIL1-chromatin binding would be altered in OGG1 inhibitor-treated cells upon oxidative damage induction. To study NEIL1-chromatin binding, fluorescence recovery after photobleaching (FRAP) experiments were performed in U2OS cells expressing NEIL1-GFP. Menadione was used to induce oxidative stress, as it has been reported to induce an increase in 8-oxoG levels [44]. U2OS cells co-treated with menadione and TH5487 displayed a significant reduction in NEIL1 nuclear mobility compared to control cells (Figure 3a–c) indicating that NEIL1-chromatin binding is increased upon OGG1 inhibition.

### 2.4. NEIL1 Retention Is Increased in a Dose-Dependent Manner upon DNA Damage Induction and TH5487 Treatment

**Figure 4:**
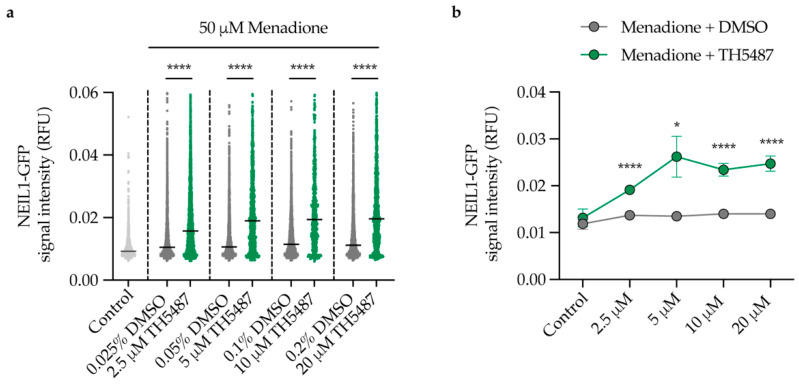
Dose-dependent retention of NEIL1 upon inhibition of OGG1. (**a**,**b**) NEIL1-chromatin binding. U2OS cells expressing NEIL1-GFP were exposed to the indicated treatment conditions. In situ extraction with 0.1% triton X-100 for 1 min was performed prior to fixation with 4% paraformaldehyde and NEIL1-GFP nuclear fluorescence signal intensity was quantified. RFU, relative fluorescence units. Individual cell data with median values are shown in (**a**) and mean values in (**b**). Scatter plots show data of at least 500 cells per condition. Data are median (**a**) or mean ± SEM (**b**) of two independent experiments with two technical repeats each. Statistical significance was determined using unpaired, two-sided *t*-test (* *p* < 0.05, **** *p* < 0.0001).

Having shown that NEIL1 is strongly recruited and bound to DNA lesions upon OGG1 inhibition, we hypothesized that TH5487 treatment should result in a dose-dependent increase in NEIL1 retention in oxidatively stressed cells. Indeed, using an in situ extraction assay, we found that significantly more NEIL1-GFP was retained in the nucleus of cells with increasing doses of TH5487 (Figure 4a,b). These data suggest that oxidative damage that cannot be repaired by catalytically inhibited OGG1 is increasingly recognized and bound by NEIL1.

### 2.5. TH5487 Treatment Results in Prolonged Accumualtion of NEIL2-GFP at DNA Damage Sites

**Figure 5:**
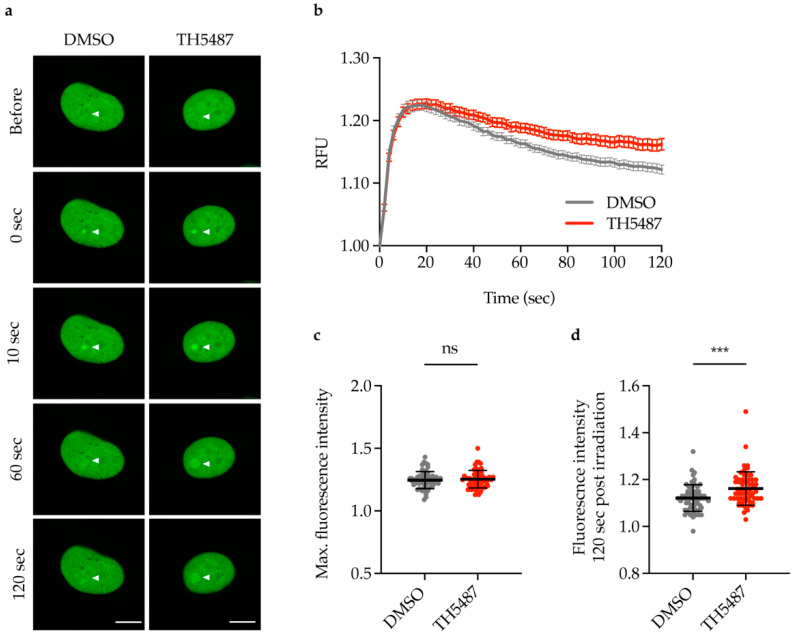
TH5487 treatment results in prolonged accumulation of NEIL2-GFP at DNA damage sites. (**a**) NEIL2 recruitment kinetics. U2OS cells expressing NEIL2-GFP were treated with either 0.1% DMSO or 10 μM TH5487 for 1 h, pre-sensitized with Hoechst, and irradiated with a 405 nm laser to induce DNA damage. Representative images before and after laser microirradiation at the indicated time points are shown. Arrows indicate the spots of laser irradiation. Scale bar, 10 μm. (**b**–**d**) Quantification of NEIL2-GFP recruitment kinetics (**b**), maximum fluorescence intensity (**c**) and fluorescence intensity 120 s post irradiation at the irradiated spots (**d**) in U2OS cells treated as described in (**a**). RFU, relative fluorescence units. Data are mean ± SEM (**b**) or mean ± SD (**c**,**d**) of five independent experiments. Statistical significance was determined using unpaired, two-sided *t*-test (ns, non-significant, *** *p* < 0.001).

Since there is some degree of overlapping substrate specificity between NEIL1 and NEIL2 [2,17], we sought to study NEIL2 recruitment to DNA damage sites following OGG1 inhibition. DNA damage was induced with laser microirradiation in U2OS cells constitutively expressing NEIL2-GFP. NEIL2-GFP was recruited to a similar extent in TH5487- and DMSO-treated cells as indicated by the maximum fluorescence intensity detected at the irradiated spots (Figure 5a–c). However, NEIL2 binding at the laser-induced DNA lesions was significantly prolonged in TH5487-treated cells 120 s post irradiation (Figure 5d). This prolonged accumulation suggests that NEIL2 may be involved in repair of lesions that could not be readily repaired by the inhibited OGG1 and therefore could play a potential backup role following OGG1 inhibition.

### 2.6. NEIL1 and NEIL2 Nuclear Retention Increases upon DNA Damage Induction in OGG1-Depleted Cells

**Figure 6:**
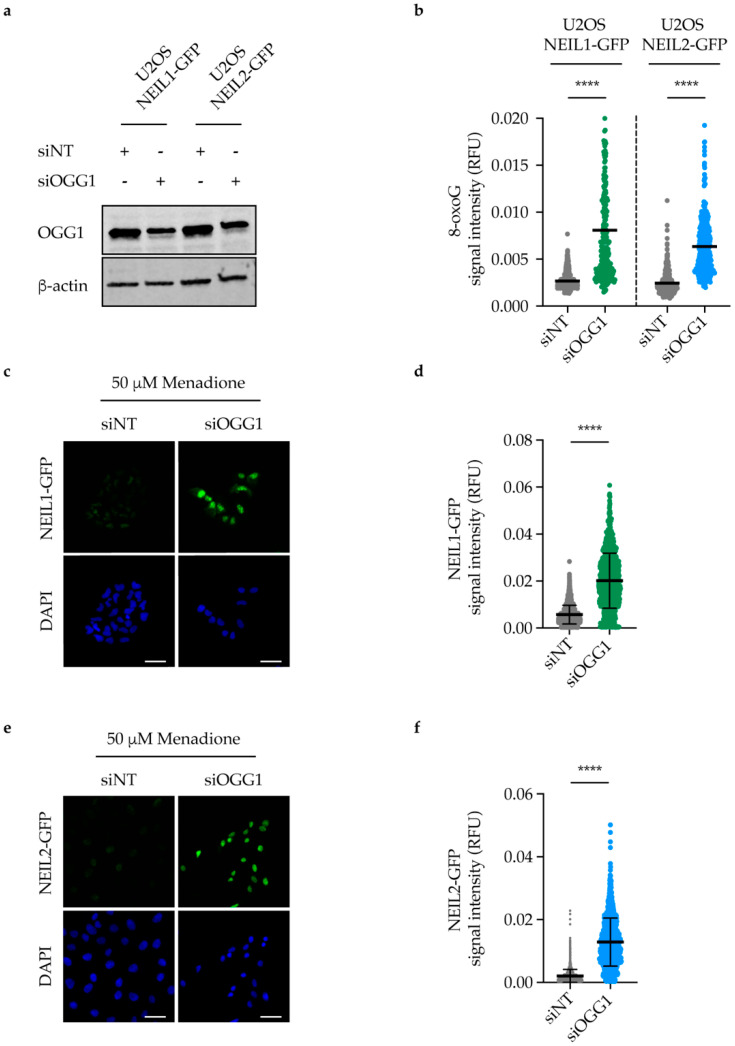
NEIL1 and NEIL2 nuclear retention increases upon DNA damage induction in OGG1-depleted cells. (**a**) Representative Western blot analysis showing OGG1 expression level in U2OS cells expressing NEIL1-GFP or NEIL2-GFP after 96 h of OGG1 siRNA transfection. (**b**) Quantification of 8-oxoG lesions after depleting OGG1 in NEIL1-GFP or NEIL2-GFP expressing U2OS cells. Cells were transfected with either 10 nM non-targeting siRNA (siNT) or siRNA targeting OGG1 (siOGG1) for 96 h. Cells were then treated with 50 μM menadione for 1 h, fixed and stained for 8-oxoG. RFU, relative fluorescence units. Scatter plot shows mean and individual data from three independent experiments. (**c**) NEIL1 nuclear retention after in situ extraction. U2OS cells expressing NEIL1-GFP were transfected with either 10 nM siNT or siOGG1 for 96 h. Cells were then treated with 50 μM menadione for 1 h followed by in situ extraction. Representative images are shown. Scale bar, 40 μm. (**d**) Quantification of NEIL1-GFP fluorescence intensity in cells treated as described in (**c**). RFU, relative fluorescence units. (**e**) NEIL2 nuclear retention after in situ extraction. U2OS cells expressing NEIL2-GFP were transfected with either 10 nM siNT or siOGG1 for 96 h. Cells were then treated with 50 μM menadione for 1 h followed by in situ extraction. Representative images are shown. Scale bar, 40 μm. (**f**) Quantification of NEIL2-GFP fluorescence intensity in cells treated as described in (**e**). RFU, relative fluorescence units. Scatter plots show mean ± SD (**d**,**f**) and individual data of three independent experiments. Statistical significance was determined using unpaired, two-sided *t*-test (**** *p* < 0.0001).

To confirm that the observed increased retention of NEIL1 and NEIL2 is not due to an off-target effect of TH5487, we depleted OGG1 using siRNA in U2OS cells expressing either NEIL1-GFP or NEIL2-GFP (Figure 6a and Figure S1). After 96 h of siRNA transfection, cells were treated with menadione to induce oxidative stress, followed by in situ extraction to wash off any unbound NEIL1/2 proteins. Notably, OGG1 knockdown led to an increase in 8-oxoG lesions in these cells (Figure 6b). Moreover, significantly more NEIL1-GFP and NEIL2-GFP were retained in the nucleus of OGG1-depleted cells compared to control cells, suggesting a potential backup role for NEIL1 and NEIL2 following OGG1 knockdown (Figure 6c–f).

### 2.7. TH5487 Leads to 8-oxoG Lesion Accumulation upon Induction of Oxidative Stress in NEIL1-or NEIL2-Depleted Cells

**Figure 7:**
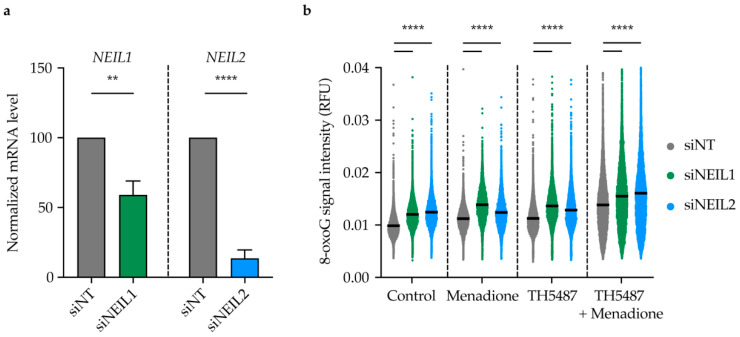
8-oxoG lesions accumulate in NEIL1- or NEIL2-depleted cells upon OGG1 inhibition and oxidative stress induction. (**a**) Normalized mRNA level of *NEIL1* and *NEIL2*. U2OS cells were transfected with either 10 nM non-targeting siRNA (siNT) or a pool of siRNA targeting NEIL1 (siNEIL1) or NEIL2 (siNEIL2). Real-time qPCR was performed 96 h post siRNA transfection. 18S rRNA was used a housekeeping control gene. Data are mean ± SD of three (NEIL1) or four independent experiments (NEIL2) with three technical replicates each. (**b**) Quantification of nuclear 8-oxoG lesions in NEIL1- or NEIL2-depleted U2OS cells. NEIL1 or NEIL2 were depleted using siRNA as described in (**a**). After 96 h of transfection, cells were incubated for 1 h in serum-free medium containing 50 μM menadione or 10 μM TH5487 or a combination of 10 μM TH5487 and 50 μM menadione. Cells were then fixed with 4% paraformaldehyde and stained for 8-oxoG. Scatter plot shows mean and individual data of at least 6000 cells for each treatment condition from two independent experiments. RFU, relative fluorescence units. Statistical significance was determined using unpaired, two-sided *t*-test (** *p* < 0.01, **** *p* < 0.0001).

In order to further characterize the role of NEIL1 and NEIL2 in OGG1 inhibitor-treated cells, we depleted NEIL1 and NEIL2 in U2OS cells using siRNA (Figure 7a). NEIL1- and NEIL2-depleted cells showed slightly enhanced survival upon treatment with TH5487 for 96 h (Figure S2a). Fewer incisions were observed in OGG1-inhibited cells after NEIL1 and NEIL2 knockdown (Figure S2b), indicating that NEIL1 and NEIL2 play a key role in initiating base excision repair after OGG1 inhibition. Interestingly, more nuclear 8-oxoG lesions were detected in NEIL1/2-depleted cells after 1 h treatment with TH5487 and menadione (Figure 7b), suggesting that NEIL1 and NEIL2 might be involved as backup glycosylases to repair oxidized guanine lesions in OGG1 inhibitor-treated cells.

## 3. Discussion

Eleven human DNA glycosylases have been identified so far with some degree of overlapping substrate specificity. Backup mechanisms within and between DNA repair pathways have been illustrated in mice knockout studies [2]. Here, our aim was to determine whether NEIL1 or NEIL2 could compensate for the enzymatic inhibition of OGG1 in mammalian cells. 8-oxoG is a pre-mutagenic lesion since it can mispair to adenine [4]. To avoid genetic transversion, OGG1 recognizes 8-oxoG opposite to cytosine in duplex DNA to initiate BER. Interestingly, homozygous *OGG1^−/−^*-deficient mice were found to be viable despite the high load of 8-oxoG detected in their genome. They were reported to display a moderately elevated spontaneous mutation rate without developing malignancies [40]. However, Sakumi et al. monitored OGG1 knockout mice for a longer period and found that lung tumorigenesis develops in these mice 1.5 years after birth [45]. Since 8-oxoG lesions are prone to further oxidation generating hydantoins which are repaired mainly by NEIL glycosylases [2,16], we sought to study whether NEIL1 or NEIL2 can compensate for OGG1’s impairment.

We employed TH5487 to pharmacologically inhibit OGG1 and studied NEIL1 recruitment kinetics and chromatin binding upon OGG1 inhibition. TH5487 is a selective OGG1 inhibitor that binds OGG1’s active site, causing accumulation of genomic 8-oxoG lesions as detected by mass spectrometry [13]. Consistently, immunostaining of 8-oxoG lesions and a modified comet assay have both demonstrated that TH5487 treatment indeed results in an increase in 8-oxoG level in DNA [42,43]. We show that TH5487 does not engage with recombinant NEIL1 (Figure 1) excluding an off-target interaction between TH5487 and NEIL1. This is consistent with our previous data, showing that TH5487 does not inhibit NEIL1’s activity in an in vitro biochemical assay [13].

Our observation that NEIL1 recruitment to DNA damage sites is significantly increased and prolonged after TH5487 treatment (Figure 2a–d) suggests that NEIL1 is recruited as a potential backup for OGG1 in OGG1 inhibitor-treated cells. The increased recruitment observed in laser-irradiated cells is further supported by our finding that NEIL1-chromatin binding is elevated upon OGG1 inhibition in cells under oxidative stress (Figure 3 and Figure 4).

Inhibition of OGG1 by TH5487 results in accumulation of genomic 8-oxoG lesions [13,42,43]. Similarly, we show here that siRNA-mediated OGG1 depletion also leads to accumulation of 8-oxoG lesions in DNA after menadione treatment (Figure 6b). 8-oxoG lesions are prone to further oxidation because of their low redox potential, giving rise to hydantoin lesions [14,16]. Since hydantoin lesions are primarily bound and repaired by NEIL1, this may explain the significant reduction in NEIL1’s mobility, and thus increased binding to damaged chromatin in TH5487-treated cells (Figure 3). Moreover, we show a TH5487-mediated dose-dependent increase in NEIL1 retention in pre-extracted cells after damage induction (Figure 4). A similar degree of NEIL1 retention was observed in OGG1-depleted cells (Figure 6c–d), demonstrating that the observed phenotype is not due to an off-target effect of TH5487.

NEIL1 and NEIL2 glycosylases have been reported to recognize and excise common substrates [2,17,33]. Laser microirradiation experiments show that NEIL2 binding to DNA damage is prolonged following OGG1 inhibition (Figure 5). Similarly, more NEIL2-GFP was retained bound to damaged chromatin in OGG1-depleted cells upon inducing oxidative stress with menadione (Figure 6e–f), suggesting that NEIL2 might also be involved as a backup glycosylase for OGG1.

The fact that NEIL1 recruitment to DNA damage sites was more affected by TH5487 (Figure 2) than NEIL2 (Figure 5) may suggest that NEIL2 plays a minor backup role for OGG1 compared to NEIL1. This is consistent with the observation that, in NEIL1-depleted cells, NEIL2 can serve as a backup for NEIL1 in pre-replicative repair of oxidized DNA lesions. It is worth noting that NEIL2’s efficiency in repairing replicating DNA was reported to be significantly lower than that of NEIL1 [25].

NEIL1 and NEIL2 depletion slightly enhanced the survival of U2OS cells after treatment with TH5487 (Figure S2a), likely due to fewer DNA incisions (Figure S2b). This suggests that NEIL1 and NEIL2 are involved in base excision repair of oxidized guanine lesions that accumulate after TH5487 treatment. Furthermore, genomic 8-oxoG lesions accumulate upon inducing oxidative stress with menadione in NEIL1/2-depleted cells. Importantly, the level of nuclear 8-oxoG is elevated after NEIL1/2 depletion in cells treated with TH5487 alone or in combination with menadione (Figure 7b), indicating a potential backup role for NEIL1 and NEIL2 following OGG1 inhibition.

Taken together, our results suggest that NEIL1, and to a lesser extent NEIL2, compensate for the impaired repair activity of OGG1 in order to maintain DNA integrity. Our study thus exemplifies potential backup within the base excision repair pathway.

## 4. Materials and Methods

### 4.1. Cell Culture and Treatments

U2OS cells were grown at 37 °C in a 5% CO_2_ atmosphere in DMEM (Gibco) growth medium supplemented with 10% of fetal bovine serum (FBS; Gibco) and 100 U/mL penicillin-streptomycin (Gibco). In order to induce DNA oxidative damage, cells at about 80% confluency were challenged with menadione sodium bisulfite (Sigma-Aldrich, St. Louis, USA, CAS 130-37-0) in serum-free DMEM for the indicated periods of time. To inhibit OGG1, 10 μM TH5487 or 0.1% DMSO (VWR chemicals, Radnor, USA) were used according to the indicated experimental scheme for the indicated time periods.

### 4.2. Differential Scanning Fluorimetry (DSF)

The DSF assay was done according to the protocol previously described by Michel et al. [46]. In Brief, 0.2 μL triplicates in a 2:1 dilution of a 10 mM TH5487 solution and a DMSO control were spotted in White BioRad 384-well plates. Next, 9.8 μL of protein buffer, containing 25 mM Tris-acetate pH 7.5, 150 mM NaCl, 10% glycerol, 1 mM DTT, 5x SYPRO Orange, and 4 μM NEIL1 lacking 56 amino acids dispensable for activity at the disordered C-Terminus, were added to each well, yielding a final protein concentration of 3.92 μM and a final compound concentration ranging from 1.56 μM to 50 μM. The plates were sealed with BioRad MicroSeal ‘B’, centrifuged at 1000 rpm and subjected to a temperature gradient of 20 to 95 °C in a freshly started and cold Roche Light Cycler 480 II. The resulting fluorescence at 465–580 nm with an excitation wavelength of 465 nm was measured. Visual quality control of each graph was performed, and the resulting data were processed and imported to a GraphPad Prism template provided by Niesen et al. [47].

### 4.3. NEIL1-GFP and NEIL2-GFP Plasmids Construction

To generate NEIL1 and NEIL2 overexpression vectors, human NEIL1 and NEIL2 coding sequences excluding the stop codon were cloned into the SalI-XhoI restriction sites of pENTR1A no CCDB (w48-1) vector (Addgene plasmid #17398), which had been modified to contain EGFP in the XhoI-XbaI site (a kind gift from Dr. P. Herr). Following sequence verification, entry clones were shuttled using Gateway cloning LR clonase reaction (Thermo fisher scientific) into pLENTI-CMV Puro DEST vector (w118-1) (E. Campeau, Addgene plasmid #17452). Kanamycin was used to select putative positive clones. Destination vectors were verified by sequencing. U2OS cells were then transfected with destination constructs using jetPEI (Polyplus) and selected with 1 μg/mL puromycin for 10 days. To minimize variability in expression levels among the different positive clones, clonal expansion was carried out to generate a single clone of U2OS cells, constitutively expressing C-terminus GFP-tagged NEIL1 or GFP-tagged NEIL2.

### 4.4. Live Cell Microscopy, Laser Microirradiation and Fluorescence Recovery after Photobleaching

Live cell microscopy, microirradiation and FRAP experiments were done as previously described by Hanna et al. [42]. Briefly, U2OS cells constitutively expressing NEIL1-GFP or NEIL2-GFP were seeded on µ-Grid ibidi dishes (35 mm with grid, ibidi #81166) 24 h prior to the indicated treatment. For laser microirradiation, 10 μg/mL Hoechst were employed to pre-sensitize the cells for 10 min at 37 °C. In order to minimize background fluorescence, the DMEM culture medium was replaced by live cell imaging medium (Thermo Fisher Scientific, Waltham, MA, USA, Catalog number: 31053028) supplemented with 10% FBS, penicillin-streptomycin antibiotics and 25 mM HEPES. Cells were treated with either 10 μM TH5487 or 0.1% DMSO for 1 h. Live cell imaging was done at Zeiss LSM780 confocal microscope equipped with a temperature-adjustable environmental chamber and a UV-transmitting Plan-Apochromat 40×/1.30 Oil DIC M27 objective. The circular region tool of the ZEN software (ZEN, Zeiss, Oberkochen, Germany) was used to select a nuclear spot of a 10-pixel diameter. DNA damage was induced at the selected spot using a 405 nm diode laser with intensity set at 100% (spot irradiation, zoom 5, pixel dwell time 12.61 μs, 1 iteration). In order to quantitatively evaluate the recruitment kinetics of NEIL1-GFP or NEIL2-GFP, fluorescence intensity at the irradiated spot was recorded and normalized to the pre-irradiation level. Background fluorescence and loss of fluorescence over the time course of the experiment were accounted for. Images were processed in ImageJ. Data of 90 nuclei from six independent laser microirradiation experiments for NEIL1 or 60 nuclei from five independent microirradiation experiments for NEIL2 were averaged. The mean curve, standard error of the mean and standard deviation were calculated and displayed using GraphPad Prism.

To induce oxidative stress for FRAP experiments, cells were challenged with 50 μM menadione sodium bisulfite (Sigma-Aldrich, St. Louis, MO, USA, CAS 130-37-0) dissolved in live cell imaging medium supplemented with penicillin-streptomycin antibiotics, 10% FBS and 25 mM HEPES (Gibco) as a pH buffer. Then, 0.1% DMSO or 10 μM TH5487 were added together with the oxidant to the cell medium for 1 h. The FRAP assay was performed according to the protocol described by Hanna et al. [42]. Live cell imaging was done at a Zeiss LSM780 confocal microscope using a Plan-Apochromat 63x/1.4 Oil DIC M27 objective. Images were processed in ImageJ. Data from three independent FRAP experiments were averaged. The mean curve, standard error of the mean and standard deviation were calculated and displayed using GraphPad Prism.

### 4.5. Quantitative Microscopy

For quantitative microscopy, cells were seeded on 96-well plates (Corning 4680) at a density of 10,000 cells/well. Cells were co-treated with 50 μM menadione and increasing concentrations of TH5487 or equivalent amounts of DMSO in serum-free DMEM for 1 h. Cells were pre-extracted with 0.1% Triton X-100 in PBS for 1 min, fixed in 4% paraformaldehyde for 20 min. Images were taken using an Image Xpress Micro (Molecular Devices, San Jose, CA, USA) microscope with a 20× lens.

8-oxoG staining was done according to the protocol previously described by Hanna et al. [42]. Briefly, cells were fixed in 4% paraformaldehyde for 20 min, permeabilized with 0.5% Triton X-100 in PBS for 5 min and treated with RNAse buffer (1 mM Ethylenediaminetetraacetic acid (EDTA), 10 mM Tris-HCl (pH 7.5), 0.4 mM NaCl, and 100 μg/mL RNAse (Zymo Research)) for 1 h at 37 °C. This was followed by treating the cells with 2.5 N HCl for 30 min at room temperature to unwind the DNA duplex. For neutralization, cells were then treated with 0.1 M sodium borate Na_2_Bo_4_O_7_ (pH 8.8) for 10 min and blocked with 4% bovine serum albumin (BSA; Sigma) in PBS for 1 h. Cells were then incubated overnight at 4 °C with anti-8-OHdG antibody (Abcam, Cambridge, UK, AB48508, N45.1) at 1:400. Alternatively, cells were permeabilized, blocked as previously mentioned and stained for γH2AX using anti-γH2AX antibody (Cell signaling 2577S) at 1:1000. Cells were incubated with secondary antibodies Alexa Fluor 647 or Alexa Fluor 488 (Thermo Fisher Scientific, Waltham, MA, USA) for 1 h at room temperature. DNA was stained with 4′,6-diamidino-2-phenylindole (DAPI). Images were acquired using Zeiss LSM780 confocal microscope at 20× magnification or an Image Xpress Micro (Molecular Devices, San Jose, CA, USA) microscope with a 20× lens. Fluorescence intensities per cell nucleus were determined using a pipeline generated in Cell Profiler software (Broad Institute) and plotted using GraphPad Prism.

### 4.6. OGG1 Knockdown and In Situ Extraction

Fifty thousand U2OS cells expressing NEIL1-GFP or NEIL2-GFP were seeded on µ-Grid ibidi dishes (35 mm with grid, ibidi #81166) and transfected with either 10 nM non-targeting siRNA (siNT) or siRNA targeting OGG1 (siOGG1) using INTERFERin (Polyplus Transfections, New York, NY, USA) according to the protocol provided by the manufacturer. Allstars Negative control siRNA (Qiagen, Hilden, Germany, catalogue number 1027280) was used as a negative control. The sequence of siOGG1 is 5′-CGGAUCAAGUAUGGACACUGA-3′.

Then, 96 h post transfection, cells were treated with 50 μM menadione for 1 h. In situ extraction was done using 0.1% Triton X-100 in PBS for 1 min followed by fixation in 4% paraformaldehyde for 20 min. Images were acquired using a Zeiss LSM780 confocal microscope at 20× magnification. Fluorescence intensities of NEIL1-GFP or NEIL2-GFP within cell nucleus were analyzed from three independent experiments using Cell Profiler software (Broad Institute) and displayed using GraphPad Prism.

### 4.7. Western Blot

Cells transfected with siNT or siOGG1 were washed in PBS and lysed in RIPA buffer (50 mM Tris–HCl pH 7.5, 1 mM EDTA, 150 mM NaCl, 0.5% sodium deoxycholate, 1% NP-40 and 0.1% SDS supplemented with complete protease inhibitor cocktail (04693116001, Roche). After sonication at 0.7 cycle and 70% amplitude using UP100H, ultrasonic processor (Hielscher Ultrasonics, Teltow, Germany), protein concentration in the lysates was measured using the Pierce BCA Protein Assay Kit (ThermoFisher Scientific). Equal amounts of protein were treated with Laemmli Sample Buffer (BioRad), heated to 90 °C and then loaded on Mini-PROTEAN precast gels (Bio-Rad, Hercules, USA) running at 150 V. Blotting was done by transferring the separated proteins to a nitrocellulose membrane (Bio-Rad) using Trans-Blot Turbo Transfer System (Bio-Rad). For blocking, membranes were incubated in blocking buffer (5% skimmed milk in TBS supplemented with 0.05% Tween-20 (TBS-T)) at room temperature for 1 h followed by incubation with primary antibodies overnight at 4 °C and secondary antibodies diluted at 1:10,000 at room temperature for 1 h. The following primary antibodies were used: rabbit anti-OGG1 (ab124741, Abcam) at 1:1000 and mouse anti-Actin (ab6276, Abcam) at 1:10,000. All antibody dilutions were prepared using the blocking buffer. An Odyssey Fc Imager was used to visualize bands. Uncropped Western blots are shown in Supplementary Figure S1.

### 4.8. NEIL1 and NEIL2 Knockdown

U2OS cells were seeded on 96-well plates (Corning 3904) at a density of 5000 cells/well, transfected with siRNA and incubated with increasing doses of TH5487 ranging from 1.5 μM to 50 μM for 96 h. INTERFERin (Polyplus Transfections) was used according to the manufacturer’s protocol to transfect cells with either 10 nM non-targeting siRNA (siNT) or a pool of siRNA targeting NEIL1 (siNEIL1, FlexiTube GeneSolution GS79661, Qiagen, catalogue number 1027416) or pool of siRNA targeting NEIL2 (siNEIL2, FlexiTube GeneSolution GS252969, Qiagen, catalogue number 1027416). Allstars Negative control siRNA (Qiagen, catalogue number 1027280) was used as a negative control.

### 4.9. Cell Viability Assay

Resazurin (R7017, Sigma Aldrich) was used at a final concentration of 0.01 mg/mL to assess the viability of NEIL1/2-depleted cells that have been seeded on 96-well plates (Corning 3904) and pretreated with increasing doses of TH5487. After 6 h of incubation with resazurin, a Hidex Sense plate reader was used to measure fluorescence at ex530/em590.

### 4.10. Real-Time PCR

RNA was isolated from cells using TRIzol reagent (Invitrogen, Carlsbad, USA, catalogue number 15596018) according to the manufacturer’s protocol. The quantity and quality of the isolated RNA was determined using NanoDrop 8000 (Thermo Fisher Scientific). Quantitect Reverse Transcription Kit (Qiagen, Hilden, Germany, catalogue number 205314) was used for cDNA synthesis following the manufacturer’s protocol. PCR was done using 2.5 μL of cDNA at a concentration of 5 ng/μL mixed with 0.5 μM of forward and reverse primers and 1× of iTaq Universial SYBR Green Supermix (Bio-Rad, Hercules, USA, catalogue number 1725124) at final reaction volume of 12.5 μL. Amplification was done using a Rotor-Gene Q PCR cycler (Qiagen) with an initial holding step of 5 min at 95 °C, followed by 40 cycles of 5 s at 95 °C and 10 s at 60 °C. The relative mRNA levels of *NEIL1* and *NEIL2* were calculated using the 2ΔΔCt method after normalization to 18S rRNA expression in siNT transfected cells. The sequences of used primers are listed in Supplementary Table S1.

### 4.11. Statistical Analysis

Data from two to six independent experiments were subjected to a two-tailed Student’s *t*-test to assess the statistical significance and presented as mean or median ± SEM or SD.

## Supplementary Materials

The following are available online at https://www.mdpi.com/article/10.3390/ijms22094542/s1.
