# Supplementary material for: NEIL1 and NEIL2 Are Recruited as Potential Backup for OGG1 upon OGG1 Depletion or Inhibition by TH5487
Source: Int J Mol Sci. 2021 Apr 27;22(9):4542. doi: 10.3390/ijms22094542 (PMC8123590; doi:10.3390/ijms22094542)
Supplement: Supplementary file 1 [file ijms-22-04542-s001.zip › ijms-1107644-supplementary.pdf]

Supplementary Materials

NEIL1 and NEIL2 are recruited as potential backup for OGG1 upon OGG1 depletion or inhibition by TH5487

Bishoy M. F. Hanna[1], Maurice Michel[1], Thomas Helleday[1,2] and Oliver Mortusewicz[1*]

[1] Science for Life Laboratory, Department of Oncology-Pathology, Karolinska Institutet, 171 65 Stockholm, Sweden; bishoy.hanna@ki.se (B.M.F.H.); maurice.michel@ki.se (M.M.); thomas.helleday@ki.se (T.H.); oliver.mortusewicz@ki.se (O.M.).

[2] Weston Park Cancer Centre, Department of Oncology and Metabolism, University of Sheffield, S10 2RX Sheffield, UK.

* Correspondence: oliver.mortusewicz@ki.se

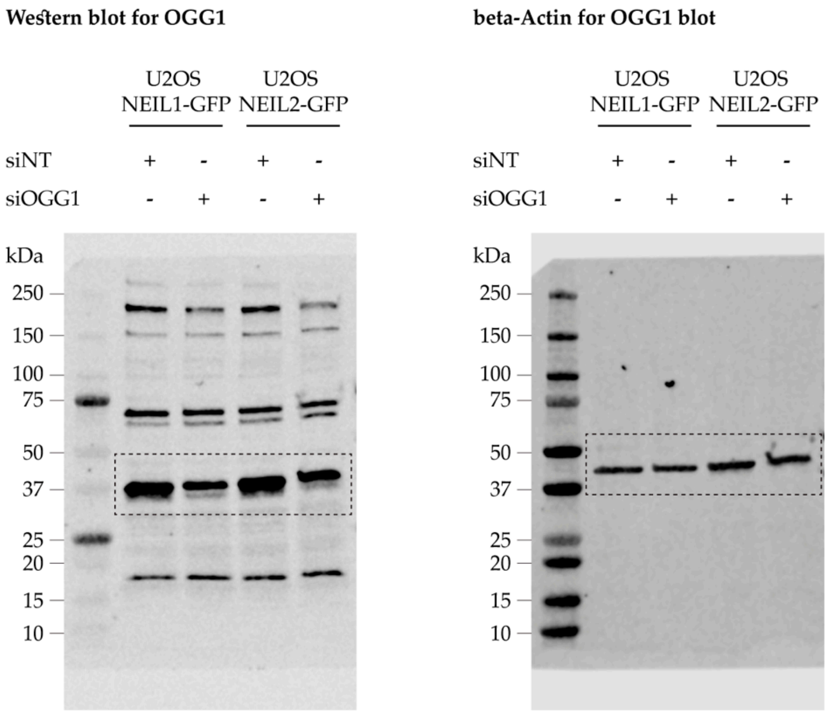

Supplementary Figure S1. Uncropped Western blots for figure 6a showing OGG1 and β-actin expression levels in U2OS cells expressing either NEIL1-GFP or NEIL2-GFP after 96 h of transfection with 10 nM non-targeting siRNA (siNT) or siRNA targeting OGG1 (siOGG1). Dotted lines indicate the cropped area.

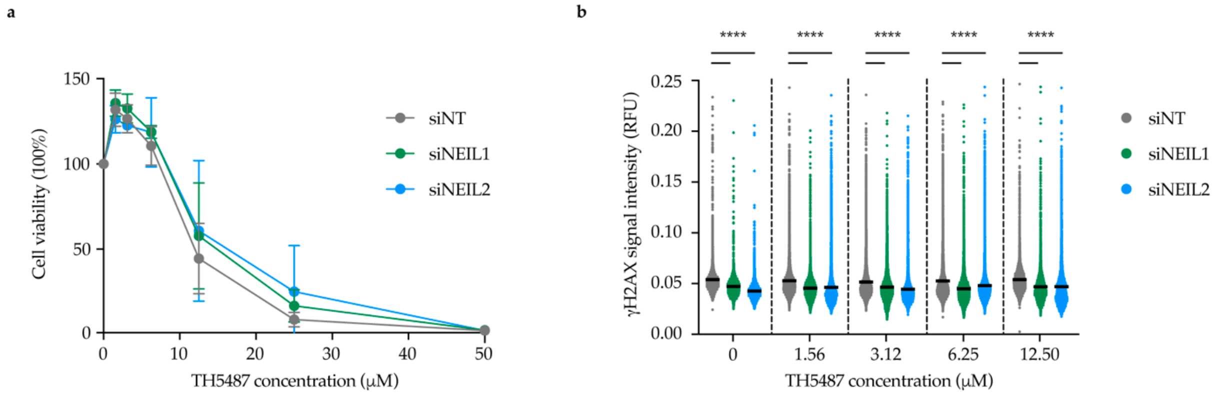

Supplementary Figure S2. (**a**) Viability of NEIL1- or NEIL2-depleted U2OS cells exposed to the indicated concentrations of TH5487 for 96 h. Data are average ±SD of eight technical replicates from four independent experiments. (**b**) Quantification of γH2AX signal intensity in NEIL1- or NEIL2-depleted cells treated with TH5487. Cells were transfected with siRNA and treated with the indicated concentrations of TH5487 for 96 h. Cells were then fixed and stained for γH2AX. Scatter plot shows mean and individual data of at least 4000 cells for each treatment condition from two independent experiments. RFU, relative fluorescence units. Statistical significance was determined using unpaired, two-sided *t*-test (**** p <0.0001).

Supplementary Table S1. qPCR primers used:

Target	Sequence 5′ to 3′	
NEIL1	Forward primer:<br>Reverse primer:	GCATTGGCAACTATCTGCGG<br>TTCTTTTTGCGGGACTTGCG
NEIL2	Forward primer:<br>Reverse primer:	CTCTTCCCCAGTGGTCACAC<br>AACGCCTCCTTCATGACCTG
18S rRNA	Forward primer:<br>Reverse primer:	AGTCCCTGCCCTTTGTACACA<br>GATCCGAGGGCCTCACTAAAC
